# Carbapenem Resistance Conferred by OXA-48 in K2-ST86 Hypervirulent *Klebsiella pneumoniae*, France

**DOI:** 10.3201/eid2607.191490

**Published:** 2020-07

**Authors:** Racha Beyrouthy, Guillaume Dalmasso, Aurélien Birer, Frédéric Robin, Richard Bonnet

**Affiliations:** Institut National de la Santé et de la Recherche Médicale, Clermont-Ferrand, France (R. Beyrouthy, G. Dalmasso, F. Robin, R. Bonnet);; Centre National de Référence de la Résistance aux Antibiotiques, Clermont-Ferrand (R. Beyrouthy, A. Birer, F. Robin, R. Bonnet);; Centre Hospitalier Universitaire, Clermont-Ferrand (R. Beyrouthy, F. Robin, R. Bonnet);; Institut National de la Recherche Agronomique (USC-2018), Clermont-Ferrand (R. Beyrouthy, F. Robin, G. Dalmasso, R. Bonnet) ;; Université Clermont Auvergne, Clermont-Ferrand (G. Dalmasso, F. Robin, R. Bonnet)

**Keywords:** β-lactamase, OXA-48, hypervirulent, Klebsiella pneumoniae, next-generation sequencing, France, antimicrobial resistance, bacteria

## Abstract

We recovered 2 carbapenem-resistant K2-ST86 hypermucoviscous *Klebsiella pneumoniae* isolates from patients in France. The isolates had genetic attributes of hypervirulent *K. pneumoniae* but differed in ability to cause mouse lethality. Convergence of hypervirulent *K. pneumoniae* toward resistance could cause a health crisis because such strains could be responsible for severe and untreatable infections.

*Klebsiella pneumoniae* is a threat to human health because of the emergence of hypervirulent *K. pneumoniae*, which has caused severe community-acquired infections, and classical multidrug-resistant *K. pneumoniae* involved in hospital outbreaks (*1*). Classical *K. pneumoniae* generally lacks the virulence genes associated with invasive diseases (*1*) and belongs to successful clonal groups, such as sequence type (ST) 11 and ST258 (*2*). Most hypervirulent *K. pneumoniae* isolates, which are mainly found in Asia (*3*,*4*), belong to the K1 and K2 capsular serotypes and are restricted to clonal complexes different from classical multidrug-resistant *K. pneumoniae* groups, such as K1-ST23, the most prevalent group (*2*). They rarely harbor acquired antimicrobial resistance genes but have virulence loci and a hypermucoviscous phenotype (*5*). We describe 2 hypermucoviscous K2-ST86 *K. pneumoniae* (positive string test) resistant to carbapenems isolated in northern and southern France.

## The Study

In 2017, we recovered the Kpn154 strain from the urine of a 35-year-old man with community-acquired urinary tract infection. He had fever (39°C) before local symptoms suggesting urinary tract infection caused by bacteremic spread, which was successfully treated with intravenous ceftriaxone. A second strain, Kpn2166, was hospital-acquired and recovered from the feces of a 70-year-old man in the intensive care unit of the hospital at which the 35-year-old patient was seem. Neither patient reported travel during the past 4 years. Both strains were resistant to all penicillins and their combinations with β-lactamase inhibitors, and to carbapenems according to EUCAST (European Committee on Antimicrobial Susceptibility Testing) guidelines (https://www.eucast.org) (Table). In addition, Kpn2166 was resistant to the third-generation cephalosporins, quinolones and tigecycline.

**Table:** Characteristics of carbapenem and hypervirulent *Klebsiella pneumoniae* isolates from 2 patients, France, 2017*

Characteristic	**Kpn154 strain**	**Kpn2166 strain**
Patient age, y/sex	35/M	70/M
Sample, context	Urine, community-acquired UTI	Feces, hospital-acquired intestinal carriage
MIC, μg/mL		
Ertapenem	2	32
Imipenem	10	10
Meropenem	2	4
Ceftazidime	0.125	>256
Ceftriaxone	0.5	>256
Cefotaxime	0.5	>256
Cefepime	0.25	>256
Aztreonam	0.06	>256
Temocillin	256	32
Tigecyclin	1	4
Colistin	0.5	0.5
Genome size, sequencing depth	5,555,907 bp, 120×	5,649,836 bp, 145×
Genotype	K2-ST86	K2-ST86
Resistance replicon, bp	IncL, 100,326	IncN, 61,761
Resistance marker	*bla*_OXA-48_	*bla*_CTX-M-15_, Δ*ompK36*, Δ*ramR*
Virulence replicon	IncHI1B/IncFIB, 215,306	IncHI1B/IncFIB, 226,677
Capsule regulator	*rmpA.2*, *rmpA2*†	rmpA.2, Δ*rmpA2*‡
Aerobactin-ST§	AbST1: *iucA1B1C1D1iutA1*	AbST1: *iucA1B1C1D1iutA1*
Salmochelin-ST§	SmST1: iroB1C1D1N1	SmST1: iroB1C1D1N1
Yersiniabactin-ST§	*ICEKp3-*YbST202LV	*ICEKp12*like- YbST13LV

*K, capsular genotype; ST, sequence type; UTI, urinary tract infection. †New *rmpA2* allele. ‡Truncated allele harboring a frameshift mutation at base 222. §Genotyped based on Kleborate schemes.

We obtained the isolates’ whole-genome sequence by hybrid de novo assembly of short and long reads generated with technologies from Illumina (https://www.illumina.com) and Oxford Nanopore (https://nanoporetech.com; European Nucleotide Archive at EMBL-EBI under accession no. PRJEB34867). We typed the isolates as K2-ST86 from whole-genome sequencing using the Institut Pasteur multilocus sequence typing scheme (https://bigsdb.pasteur.fr) and Kleborate (*6*). Kpn154 harbored carbapenemase-encoding gene *bla*_oxa-48_ and Kpn2166 the extended-spectrum β-lactamase–encoding gene *bla*_CTX-M-15_ as the only acquired β-lactamase–encoding genes. CTX-M-15 associated with the truncation of the outer membrane protein OmpK36 caused by 11-bp deletion was probably responsible for carbapenem resistance in Kpn2166. In the absence of *rpsJ* variants previously associated with tigecycline resistance, Kpn2166 resistance to tigecycline and quinolones probably resulted from a frameshift mutation in *ramR* (C→T at position 364) (*7*).

We identified IncL replicon in Kpn154 and IncN replicon in Kpn2166. The IncL replicon of Kpn154 was a canonical pOXA-48–like plasmid encoding *bla*_OXA-48_ (GenBank accession no. JN626286). In the Kpn2166 isolate, *bla*_CTX-M-15_ was encoded by a new ST9-IncN plasmid, included in an IS*26*-based composite transposon and downstream a truncated IS*Ecp1* insertion sequence.

Each isolate harbored an IncHI1B/IncFIB replicon, designated pVIR-Kpn154 and pVIR-Kpn2166 (Table), typified by the reference hvKP virulence plasmid pLVPK (*8*). They shared with pLVPK 97% pairwise identity overall and all virulence genes, including the *rmpA* and *rmpA2* genes involved in the hypermucoid phenotype (*8*). We typed the *rmpA* genes as allele 2 according to the Institut Pasteur scheme. However, Kpn2166 *rmpA2* harbored in addition a frameshift mutation (coding sequence [CDS] position 196) and 2 other mutations in virulence genes encoding the siderophores (Appendix Figure 1).

**Figure 1:**
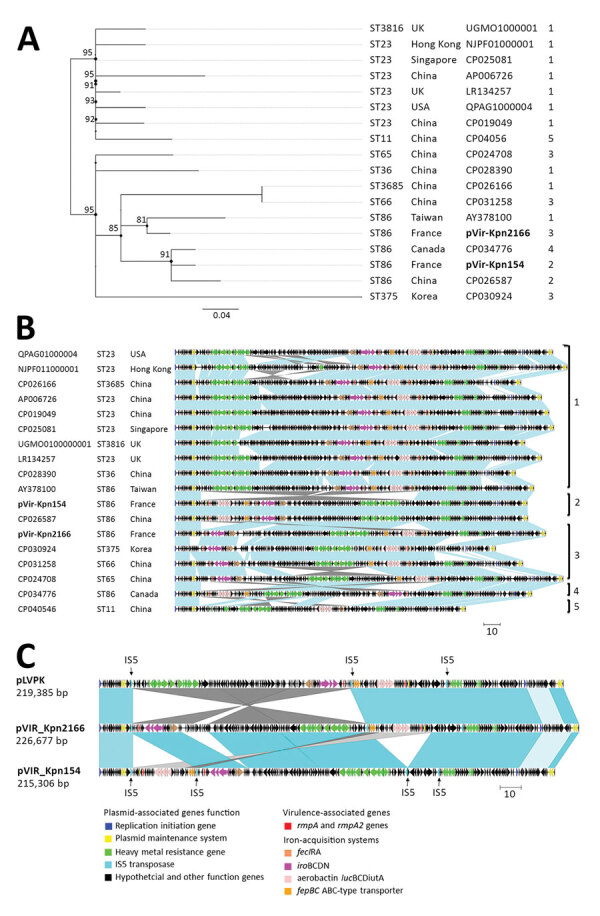
Comparison of pVIR-Kpn2166 and pVIR-Kpn154 *Klebsiella pneumoniae* isolates from 2 patients in France (bold) with 16 hypervirulent *K. pneumoniae* virulence plasmids recovered from the PATRIC database (http://www.patricbrc.org). A) Single-nucleotide polymorphism–based phylogenetic tree built by RaxML from an alignment generated by Burrows-Wheeler Aligner and filtered to remove recombination using Gubbins as previously described (*9*). The ST and the geographic origin of bacterial hosts are shown. Scale bar indicates mean number of nucleotide substitutions per site. B) Synteny analysis of hypervirulent *K. pneumoniae* virulence plasmids based on data from blastn (https://blast.ncbi.nlm.nih.gov/Blast.cgi). Virulence-based synteny groups are indicated and the operons encoding the virulence factors, virulence synteny groups of plasmids and the ST and the geographic origin of bacterial hosts. Scale bar indicates kbp. C) Comparison of the details of rearrangements observed in pVIR-Kpn2166 and pVIR-Kpn154 and in pLVPK. Scale bar indicates kbp. ST, sequence type.

We compared the pVIR-Kpn154 and pVIR-Kpn2166 plasmids with 16 complete hypervirulent *K. pneumoniae* plasmid sequences (Appendix Table) from the PATRIC database (http://www.patricbrc.org). A single-nucleotide polymorphism–based phylogenic tree showed a link between the major tree branches and the sequence types of hypervirulent *K. pneumoniae* owners but not with their geographic origin (Figure 1, panel A). This finding suggests emergence was more likely caused by spread over distant geographic areas than by local expansion and that limited horizontal transfers between hypervirulent *K. pneumoniae* isolates probably resulted from the absence of known genes involved in conjugation. Phylogenetic tree analysis also showed 5 groups based on virulence gene synteny, with the predominant group typified by pLVPK (Figure 1, panel B). Because plasmids of ST23-like hypervirulent *K. pneumoniae* all share a similar synteny of virulence genes, ST86 hypervirulent *K. pneumoniae* contains a diversity of plasmid synteny groups (Figure 1, panel A), suggesting that rearrangements of virulence genes occurred several times along plasmid evolution at rearrangement hotspots active in non-ST23 genetic background. For example, pVIR-Kpn2166 and pVIR-Kpn154 differed from reference plasmid pLVPK by the permutations in the ≈100-kb region flanked by IS5 mobile elements (Figure 1, panel C). pVIR-Kpn154 contained an additional copy of IS5, which was associated with another ≈30-kb permutation + translation event, suggesting that IS5 is a key factor in the evolution and diversity of hypervirulent *K. pneumoniae* plasmids.

The chromosome of Kpn154 and Kpn2166 exhibited similar organization but differed by 128 insertion/deletion mutations and 1,928 single-nucleotide variants (Appendix Figures 2, 3). The Kpn154 chromosome-mediated *ybt* virulence locus, which encodes the yersiniabactin, was located in the integrative conjugative element ICE*Kp3* and was typed ybST202–1LV (*6*). In Kpn2166, *ybt* was located in an original isoform of ICE*Kp12*, presenting an ≈34-kb deletion compared to the canonical 97,771-bp ICE*Kp12*, and was typed ybST13–1LV (*6*).

**Figure 2:**
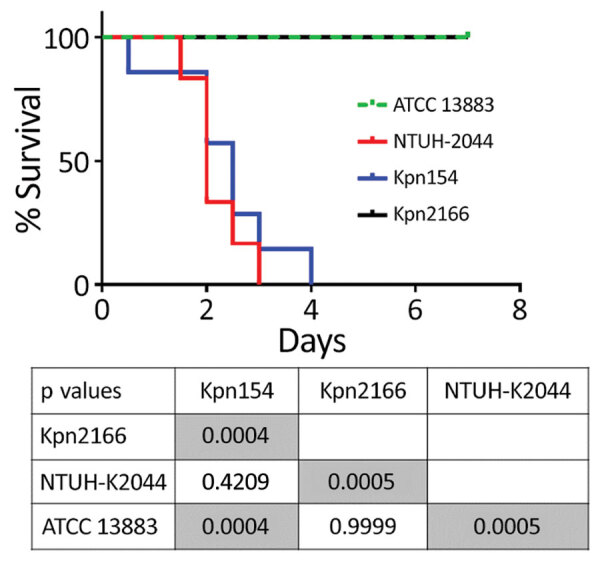
Kaplan–Meier survival curves of mice intraperitoneally challenged with *Klebsiella pneumoniae* strains Kpn154 and Kpn2166 from 2 patients in France, virulent strain NTUH-K2044, and nonvirulent ATCC 13883 strain, as previously described (*10*). Mice were injected with 10^3^ CFUs and monitored for 96 h. p values were calculated from the Mantel-Cox log rank test for survival curve comparison. Gray shading indicates significant values. ATCC, American Type Culture Collection.

Although Kpn154 and Kpn2166 have the same genetic background and share the same virulence score (*6*), they also have allelic and synteny differences in virulence genes. We therefore compared the virulence of these isolates in a sepsis model based on outbred mice challenged intraperitoneally, as described (Figure 2) (*10*). Mice injected with 10^3^ CFUs of Kpn154 or hypervirulent *K. pneumoniae* reference strain *K. pneumoniae* NTUH-2044 died in <72 h, in contrast to mice inoculated with Kpn2166 or American Type Culture Collection (ATCC) 13883, showing that only isolate Kpn154 is hypervirulent. Higher bacterial doses (10^6^ and 10^8^ CFUs) of ATCC13883 and Kpn2166 did not lead to mouse lethality, confirming that Kpn2166 is not hypervirulent in this model, despite harboring all genetic attributes of hypervirulent *K. pneumoniae* except a functional *rmpA2* gene and allelic variants of siderophore-encoding genes. 

We assessed the production of siderophores in Kpn154, Kpn2166 and the control strains as described (*11*). Although the siderophore production of the nonvirulent strain ATCC13883 (mean 30.2 + SD 1.8 μg/mL) was at the previously reported rate predicting hypervirulent *K. pneumoniae* phenotype (≥30 μg/mL), the other strains produced significantly higher siderophore levels (107.2 + 4.5 μg/mL to 306.7 + 20.2 μg/mL; Bonferroni-adjusted p = 0.0035 by Mann-Whitney test), with Kpn154 producing at the lower level (Appendix Figure 4).

## Conclusions

Our results show that a hypermucoviscous K2-ST86 strain can be avirulent in a sepsis mouse model and that hypervirulence cannot be clearly explained by siderophore production alone. Gene *rmpA2*, not required for the hypermucoviscosity phenotype as previously observed (*12*), might be required for hypervirulent phenotype because it is a main, but not the only, difference we observed between the ST86-K2 strains. Finally, these results highlight the importance of in vivo virulence investigation to identify hypervirulent *K. pneumoniae*, especially in the absence of an appropriate clinical scenario.

The threat of hypervirulent *K. pneumoniae* acquiring carbapenem resistance is becoming a reality in Asia, especially in China, where hypervirulence prevalence among carbapenem-resistant *K. pneumoniae* is 7.4%–15% (*5*). Most resistant isolates are non-K1/K2-ST11 and produce carbapenemase KPC-2;. they result from the transfer of the pLVPK-like plasmid into ST11 classical multidrug-resistant *K. pneumoniae* isolates, as observed in the 2 cases reported outside China (*5*). Inversely, the carbapenemase-producing isolate Kpn154 results from the transformation of K2-ST86 hypervirulent *K. pneumoniae* by plasmid encoding carbapenemase OXA-48, the most prevalent carbapenemase in France. Similar events occurred with the KPC-2 K2-ST86 isolate recently reported in Canada (*13*) and a few KPC-2 and NDM K1-ST23 cases documented in China and recently in the United States and United Kingdom (*5*,*14*,*15*). The combination of multidrug resistance and enhanced virulence has the potential to trigger the next clinical crisis and cause severe and untreatable infections in previously healthy persons.

Appendix Additional results from a study of carbapenem resistance conferred by OXA-48 in K2-ST86 hypervirulent *Klebsiella pneumoniae*, France
