# Supplementary material for: Carbapenem Resistance Conferred by OXA-48 in K2-ST86 Hypervirulent Klebsiella pneumoniae, France
Source: Emerg Infect Dis. 2020 Jul;26(7):1529–33. doi: 10.3201/eid2607.191490 (PMC7323517; doi:10.3201/eid2607.191490)
Supplement: Appendix — Additional results from a study of carbapenem resistance conferred by OXA-48 in K2-ST86 hypervirulent Klebsiella pneumoniae, France [file 19-1490-Techapp-s1.pdf]

Article DOI : https://doi.org/10.3201/eid2607.190924

Carbapenem Resistance Conferred by OXA-48 in K2-ST86 Hypervirulent *Klebsiella pneumoniae*, France

Appendix

Appendix Table. Features of hypervirulent *Klebsiella pneumoniae* virulence plasmids and hosts

Plasmid name	Size, bp	Host name	Sequence type	Country	GenBank accession no.
pVIR-Kpn2166	222,667	Kpn2166	86	France	This study
pVIR-Kpn154	215,306	Kpn154	86	France	This study
pLVPK	219,385	CG43	86	Taiwan	AY378100
p1-CP026587	215,697	NUHL30457	86	China	CP026587
pK2044	224,152	NTUH-K2044	23	China	AP006726
pRJA166b	228,613	RJA166	23	China	CP019049
pSGH10	231,583	SGH10	23	Singapore	CP025081
P_LR134257	224,165	NCTC9644	23	United Kingdom	LR134257
pJHTS007	230,906	JHTS007	23	United States	QPAG01000004
Unnamed	239,822	1088	23	Hong Kong	NJPF01000001
pVir_095132	208,166	WCHKP13F2	36	China	CP028390
pPUTH1	237,216	cr-hvkp3	65	China	CP024708
pL22-1	212,635	L22	66	China	CP031258
pKC-PI-HB1	196,000	KC-PI-HB1	375	Korea	CP030924
pF81_2	211,437	F81	3685	China	CP026166
Unnamed	213,869	NCTC9494	3816	United Kingdom	UGMO01000001
phvKP060	218,147	18CPO060	86	Canada	CP034776
pCR-HvKP5-VIR	177,694	CR-HvKP5	11	China	CP040546

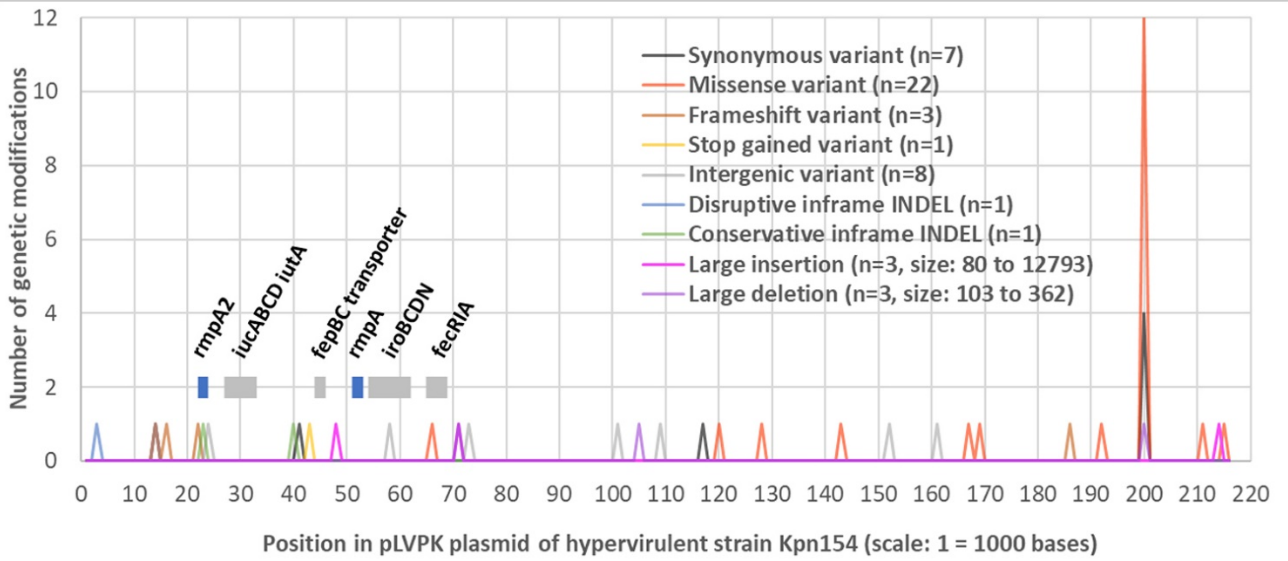

Appendix Figure 1. Comparison of genetic modifications identified in virulence plasmid of *Klebsiella pneumoniae* strain Kpn2166 with those of virulence plasmid of hypervirulent strain Kpn154. The zones encoding the virulence factors are indicated as horizontal bars (*rpmA2* and *rpmA* in blue and the siderophore-encoding zones in grey). The *rmpA2* pseudogene in strain Kpn2166 harbors a T duplication at coding sequence (CDS) position 196 leading to frameshift Tyr66fs and a GGG conservative inframe duplication at CDS position 277. The gene encoding sigma-70 family RNA polymerase sigma factor FecI harbors the missense single-nucleotide variant A→T at CDS position 62 leading to substitution His21Leu, and the enterobactin-encoding operon, an intergenic single-nucleotide variant. The zone accumulating missense and synonymous variants around position 198 kb encodes a hypothetical protein devoid of conserved domain. INDEL, insertion–deletion mutation.

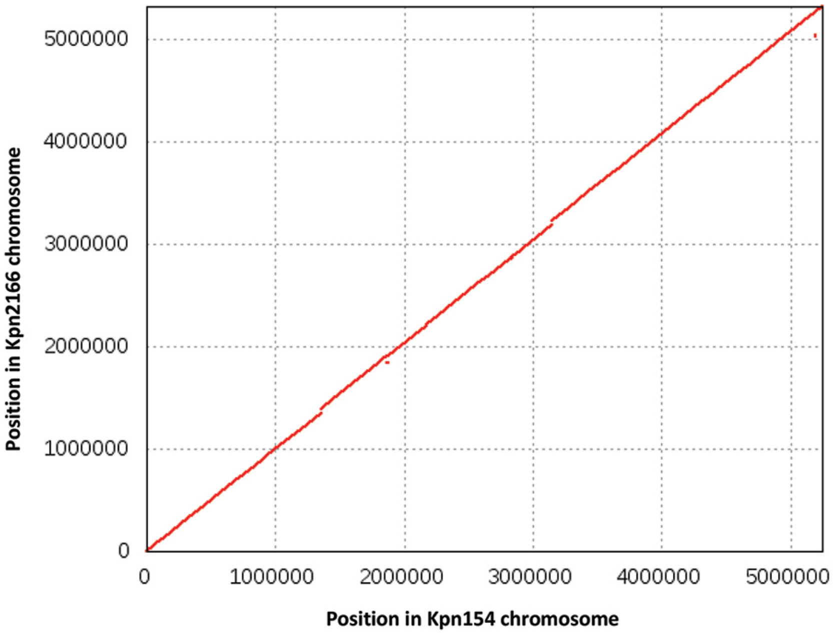

Appendix Figure 2. Dot plot comparison of Kpn154 and Kpn2166 chromosomes showing a conserved organization. The red lines indicate highly similar sequences (>95% identity) on the same strands.

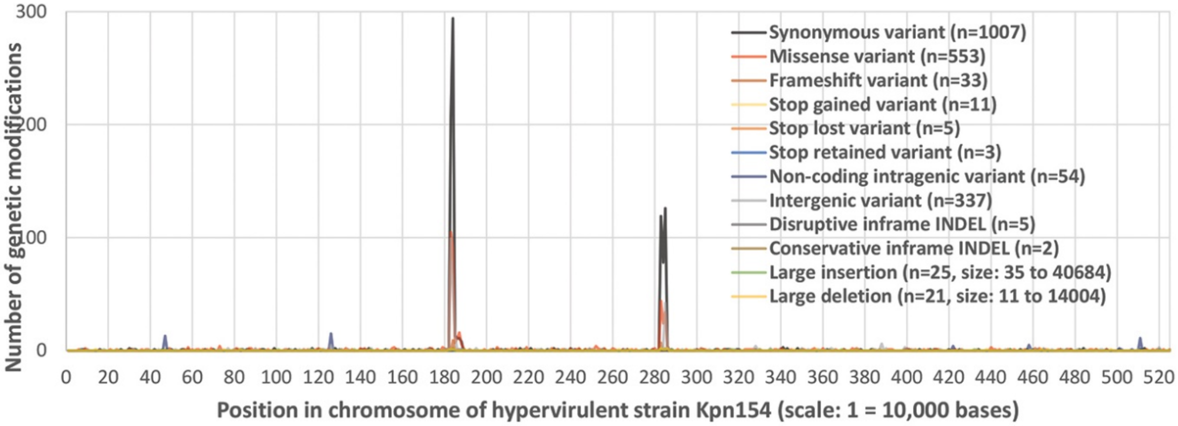

Appendix Figure 3. Comparison of genetic modifications identified in chromosome of strain Kpn2166 with those of chromosome of hypervirulent strain Kpn154. The 2 zones accumulating genetic modifications and rearrangements (positions 1,814,000–1,834,000 and 2,814,000–2,844,000) encode type IV secretion system proteins and hypothetical proteins, respectively. INDEL, insertion–deletion mutation.

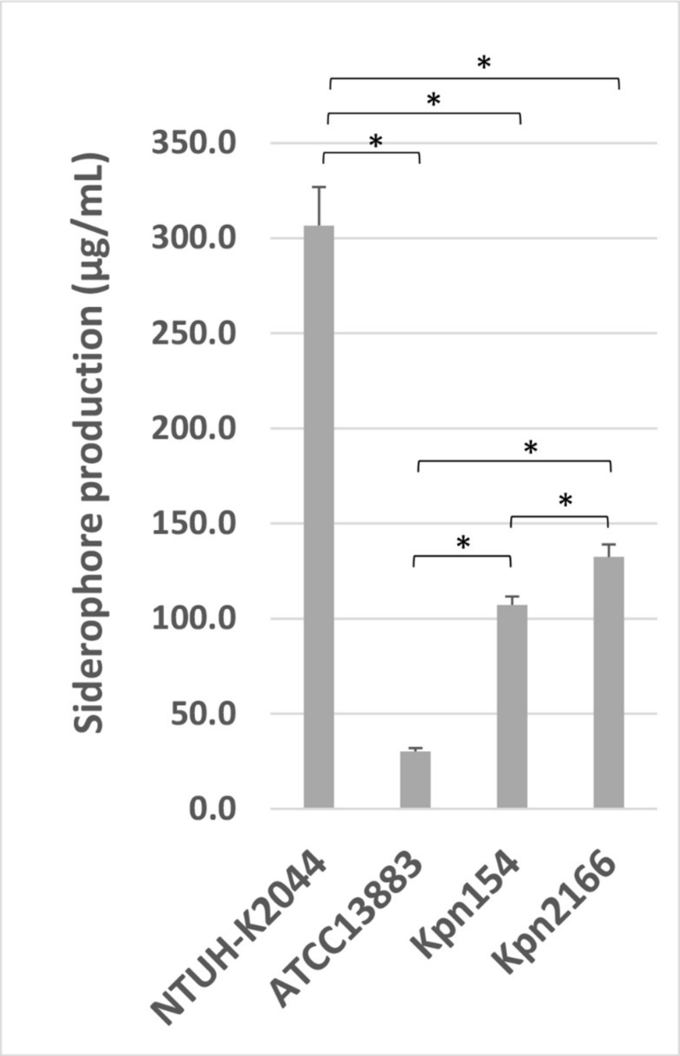

Appendix Figure 4. Estimation of siderophore production from the bacterial strains incubated overnight at 37°C in iron-chelated M9 minimal medium containing casamino acids. The siderophore concentrations were reported in µg/mL for 1×10^9 bacterial CFUs. The overall comparison performed by Kruskal-Wallis rank test showed significant differences ($p = 0.00001$). The p values for the pairwise differences reported in the figure were calculated from Mann-Whitney tests and were adjusted for multiple comparisons using Bonferroni correction. *$p<0.01$.
